# Effect of dual residual risk of cholesterol and inflammation on all-cause mortality in patients with cardiovascular disease

**DOI:** 10.1186/s12933-023-01826-3

**Published:** 2023-04-24

**Authors:** Ling Yang, Qing Yue, Fang Fang, Yinggen Zhang, Peipei Liu, Zihao Zhang, Guodong Wang, Shuohua Chen, Shouling Wu, Xiuhong Yang

**Affiliations:** 1 School of Public Health, North China University of Science and Technology, No. 21 Bohai Road, Caofeidian Eco-city, Tangshan, 063210 Hebei China; 2 Hebei Key Laboratory for Chronic Diseases, Tangshan Key Laboratory for Preclinical and Basic Research On Chronic Diseases, School of Basic Medical Sciences, North China University of Science and Technology, No. 21 Bohai Road, Caofeidian Eco-city, Tangshan, 063210 Hebei China; 3 Department of Nuclear Medicine, Kailuan General Hospital, 57 Xinhua East Rd, Tangshan, 063000 Hebei China; 4 Department of Cardiology, Kailuan General Hospital, 57 Xinhua East Rd, Tangshan, 063000 Hebei China; 5 School of Public Health, Hebei Key Laboratory for Chronic Diseases, Tangshan Key Laboratory for Preclinical and Basic Research On Chronic Diseases, School of Basic Medical Sciences, North China University of Science and Technology, No. 21 Bohai Road, Caofeidian Eco-city, Tangshan, 063210 Hebei China

**Keywords:** Low-density lipoprotein cholesterol, Hypersensitive C-reactive protein, Residual risk, All-cause mortality

## Abstract

**Background:**

Randomized controlled trials confirm that risks of residual cholesterol and residual inflammation remains in patients with cardiovascular disease (CVD) even after lipid-lowering therapy. This study aims to investigate the association between dual residual risk of cholesterol and inflammation and all-cause mortality in a real-world population with CVD.

**Methods:**

Patients with a CVD history who first took statins between 1 January 2010 and 31 December 2017 in the Kailuan Study were selected as study participants. According to low-density lipoprotein cholesterol (LDL-C) and hypersensitive C-reactive protein levels, patients were divided into those with no residual risk, residual inflammatory risk (RIR), residual cholesterol risk (RCR), and residual cholesterol and inflammatory risk (RCIR). Cox proportional hazard model was conducted to determine hazard ratio (HR) of all-cause mortality for RIR, RCR, and RCIR. Stratified analysis was conducted according to good medication adherence and 75% of the percentage LDL-C decline, high SMART 2 risk score, and blood pressure and blood glucose at standard levels.

**Results:**

After 6.10 years of follow-up, 377 all-cause deaths occurred in 3509 participants (mean age 63.69 ± 8.41 years, 86.78% men). After adjusting for related risk factors, the HR and (95% confidence interval [CI]) of all-cause mortality in the RIR, RCR, and RCIR was 1.63 (1.05, 2.52), 1.37 (0.98, 1.90), and 1.75 (1.25, 2.46), compared with no residual risk. Similar associations were observed in participants with moderate or low statin compliance, a lower percentage of LDL-C decline, high SMART 2 risk score, uncontrolled blood pressure, and uncontrolled blood glucose, in the RCIR had a 1.66-fold, 2.08-fold, 1.69-fold, 2.04-fold, and 2.05-fold higher risk of all-cause mortality, respectively, than the reference.

**Conclusion:**

Risks of residual cholesterol and residual inflammation remain in patients with CVD after receiving statins, and their combined effect significantly increases the risk of all-cause mortality. Here, this increased risk was dependent on statin compliance, LDL-C reduction, SMART 2 risk score, and blood pressure and blood glucose control.

**Supplementary Information:**

The online version contains supplementary material available at 10.1186/s12933-023-01826-3.

## Introduction

In 2020, approximately 19 million people worldwide died of cardiovascular disease (CVD), an increase of 18.7% over 2010 [1]. CVD is also the leading cause of mortality in China, accounting for 40% of the total mortality [2]. Lipid-lowering therapy is an essential measure for the secondary prevention of CVD [3], and anti-inflammatory therapy can also substantially reduce the morbidity and mortality of major adverse cardiovascular events (MACE) in patients with CVD [4]. Several studies have shown that lowering the levels of low-density lipoprotein cholesterol (LDL-C) and hypersensitive C-reactive protein (hs-CRP) can significantly reduce the risk of recurrence and mortality after the onset of CVD [5–8].

Residual cholesterol, represented by LDL-C, and residual inflammation, represented by hs-CRP, remain risk factors for the recurrence of cardiovascular events and mortality, even after patients have received aggressive lipid-lowering therapy. Studies have found that in people with a history of CVD and lipid-lowering treatment, the risk of recurrent cardiovascular events increases with increased LDL-C levels [9]. Patients treated with percutaneous coronary intervention or those with coronary heart disease who have taken statins, as well as patients with LDL-C < 1.8 mmol/L but hs-CRP > 2.0 mg/L, still have a higher risk of CVD or all-cause mortality [10–12]. These studies suggest that lipid-lowering therapy does not have a complete protective effect in patients with CVD, and residual cholesterol and residual inflammation risk can increase the risk of recurrent CVD or mortality, which are likely to be key factors affecting health outcomes [13].

A randomized controlled trial (RCT) confirmed that active lipid-lowering and anti-inflammatory therapies are equally crucial in secondary prevention of CVD [6]. However, more studies are needed to assess the association of residual cholesterol and residual inflammation with outcomes in a real-world population of patients with CVD. In the present study, we used data from the Kailuan Study to analyze the relationship between the dual residual risk of cholesterol and inflammation and all-cause mortality after the occurrence of CVD and treatment with statins in a community population.

## Methods

### Study design and participants

In this study, we used data from the Kailuan Study (registration number: ChiCTR-TNC-11001489). The Kailuan Study is an ongoing prospective cohort study, with individuals from the Kailuan community in Tangshan serving as the study population. The design and procedure of this study have been described in detail [14]. From 2006 to 2007, Kailuan General Hospital and its 11 affiliated hospitals conducted physical examinations and administered questionnaire surveys for active and retired employees of the Kailuan Group as baseline data, and then conducted follow-up every 2 years. All employees and retirees of the Kailuan Group are covered by basic medical insurance for urban employees, and drug treatment can be reimbursed by medical insurance [15].

Since 2010, we have collected drug use data of participants in the Kailuan Study through the chronic disease clinic. A total of 5443 patients took prescription statin drugs for the first time between 1 January 2010 and 31 December 2017 and had a history of CVD (including ischemic stroke, myocardial infarction, heart failure, revascularization therapy, and coronary heart disease) before taking these drugs. We excluded participants who did not undergo a physical examination after taking medication (n = 1371) and those who had no data for hs-CRP (n = 515) and LDL-C (n = 48) at baseline, Finally, 3509 patients (including 741 with ischemic stroke, 578 with myocardial infarction, 407 with heart failure, 1210 with revascularization, and 573 with coronary heart disease) were enrolled (Additional file 1: Figure S1).

This study was conducted in accordance with the guidelines of the Declaration of Helsinki and was approved by the Ethics Committee of Kailuan General Hospital. All study participants signed informed consent forms.

### Definition of exposure

For LDL-C and hs-CRP detection, participants fasted for at least 8 h, and 5 mL of fasting elbow venous blood was extracted during 7:00–9:00 on the day of physical examination. After centrifuging, the upper serum was collected for detection by professional testers using automatic biochemical analyzers (Hitachi 7600, Tokyo, Japan) in the central Laboratory of Kailuan General Hospital.

According to the grouping in previous studies related to residual risk [13, 16], we divided participants into four groups: those with no residual risk, LDL-C < 1.8 mmol/L and hs-CRP < 2 mg/L; residual inflammatory risk (RIR), LDL-C < 1.8 mmol/L and hs-CRP ≥ 2 mg/L; residual cholesterol risk (RCR), LDL-C ≥ 1.8 mmol/L and hs-CRP < 2 mg/L; and participants with residual cholesterol and residual inflammation risk (RCIR), LDL-C ≥ 1.8 mmol/L and hs-CRP ≥ 2 mg/L.

### Determination and follow-up of mortality

We collected mortality information by searching the medical information system of Kailuan General Hospital and its affiliated hospitals and the social security system of the Kailuan Group for each year. The time of the first physical examination after taking statins was taken as the starting point, and death was taken as the end point of follow-up until 31 December 2021.

### Determination of CVD

We used the International Classification of Diseases (ICD), Tenth Revision codes to identify cases of ischemic stroke (I63), myocardial infarction (I21), coronary heart disease (I25), and heart failure (I50). ICD, Ninth Revision, Clinical Modification codes 36.1, 00.66, 36.01, 36.02, 36.05, 36.06, 36.07 were used to identify revascularization therapy refers to coronary intervention and coronary artery bypass grafting. Every year, trained medical staff check the hospitalization diagnosis of study participants in all hospitals of the Kailuan Group and designated hospitals covered by municipal medical insurance. These staff record end-point events, and each diagnosis is confirmed by a physician according to the medical records during hospitalization.

### Data collection

Demographics (age, sex, education level), lifestyle factors (smoking, alcohol consumption, physical exercise, and salt intake), and medical history (diabetes, hypertension, cardiovascular history) were collected using a standardized questionnaire. For definitions of smoking, alcohol consumption, physical exercise, education level, and salt intake, see the previous definitions of the Kailuan Study [17]. Height, weight, and blood pressure were measured by professionally trained doctors. Body mass index (BMI) was calculated as weight (kg) divided by height squared (m^2^). Hypertension was defined as systolic blood pressure (SBP) ≥ 140 mmHg or diastolic blood pressure (DBP) ≥ 90 mmHg, the use of antihypertensive drugs, or a self-reported history of hypertension diagnosed by a doctor. Normal blood pressure was defined as SBP < 140 mmHg and DBP < 90 mmHg. Diabetes was defined as fasting blood glucose (FBG) ≥ 7.0 mmol/L, the use of hypoglycemic drugs, or self-reported history of diabetes diagnosed by a doctor. Normal blood glucose level was defined as FBG < 7.0 mmol/L. The measurement methods used for FBG, triglyceride (TG) and high-density lipoprotein cholesterol (HDL-C) were the same as those for LDL-C and hs-CRP. The percentage reduction in LDL-C (∆LDL-C%) was defined as the ratio of the difference between the most recent LDL-C measurement before baseline and the baseline LDL-C to the most recent LDL-C measurement before baseline.

Information on the use of antihypertensive, antidiabetic, and antiplatelet drugs was collected from the electronic records of the chronic disease clinic. According to the types of statin, we divided participants into hydrophilic statin users (i.e., pravastatin and rosuvastatin) and lipophilic statin users (i.e., atorvastatin, simvastatin, fluvastatin, lovastatin, and pitavastatin) [18]. Medication adherence was measured using the medication possession ratio (MPR); high adherence was categorized as MPR values of at least 80%, and low adherence was categorized as MPR values of less than 80% [19, 20].

### Statistical analysis

SAS 9.4 software (SAS Institute, Inc, Cary, NC, USA) was used for statistical analyses. All statistical tests were bilateral, and p < 0.05 was considered statistically significant. The mean ± standard deviation was used to describe the normal distribution of continuous variables, and analysis of variance was used for comparisons. Continuous variables with a skewed distribution are described using median (interquartile range) and compared using the Wilcoxon rank-sum test. Categorical variables are described using percentage and compared with the chi-square test. Cumulative incidence is calculated by life table and use log-rank to test for differences in cumulative incidence between groups. Using Schoenfeld residual method to test the proportional hazards (PH) assumption, the PH assumption was met (*P* = 0.781), a general Cox proportional risk model was adopted. No residual risk was used as the reference group to calculate the hazard ratio (HR) value and 95% confidence interval (CI) of all-cause mortality in the other three groups. Models were adjusted for sex, age, education, physical activity, smoking, alcohol and salt intake, BMI, TG, HDL-C, hypertension, diabetes, and use of blood pressure and hypoglycemic drugs. Multiple regression was used to compensate for the absence of covariates.

Taking into account the effect of statin adherence and degree of lipid-lowering on the outcome, we calculated the percentage decrease in LDL-C using the LDL-C level before medication and LDL-C at baseline, stratified according to whether adherence was good and 75% of the percentage of LDL-C decline, respectively, and we repeated the main analysis. To explore the effect of residual risk on all-cause mortality according to different degrees of CVD recurrence risk, we stratified participants by whether they had a high Secondary Manifestations of ARTerial disease (SMART) 2 risk score [21] and whether blood pressure and blood glucose were at normal levels.

Sensitivity analyses were performed to assess stability of the findings. First, participants who died within 1 year of follow-up were excluded and the data were re-analyzed. Second, the critical value of hs-CRP was changed to 3 mg/L for re-analysis. Third, the critical value of LDL-C was changed to 2.6 mmol/L for re-analysis. Fourth, for re-analysis, the critical values of hs-CRP and LDL-C were changed to 3 mg/L and 2.6 mmol/L, respectively.

In order to explore the influence of LDL-C and hs-CRP interaction on all-cause mortality, we put LDL-C, hs-CRP and their multiplicative interaction items into the COX model after adjusting for covariates. LDL-C was grouped according to 1.8 mmol/L, hs-CRP was grouped according to 2 mg/L, and relative excess risk due to interaction (RERI), attributable proportion due to interaction (AP), and synergy index (SI) were calculated (Eq see Additional file 2: Table S4) to evaluate the additive interaction.

## Results

### Baseline characteristics

In total, 3509 participants meeting the requirements were included in the analysis, with average age 63.69 ± 8.41 years, and 86.78% men. Participants were grouped according to LDL-C and hs-CRP levels, and 613 (17.47%) were classified as no residual risk, 295 (8.41%) as RIR, 1628 (46.39%) as RCR, and 973 (27.73%) as RCIR. The baseline characteristics are shown in Table 1.

**Table 1:** Characteristics According to Hypersensitive C-reactive Protein and Low-density Lipoprotein Cholesterol Levels

Variables	No residual risk	RIR	RCR	RCIR	*p* value
Participants, n	613	295	1628	973	
Age, years	63.23 ± 8.46	63.10 ± 8.40	63.79 ± 8.39	64.00 ± 8.41	0.18
Men, n (%)	552 (90.0)	271 (91.9)	1399 (85.9)	823 (84.6)	< 0.01
BMI, kg/m^2^	25.75 ± 3.20	26.10 ± 3.43	25.77 ± 3.12	26.42 ± 3.29	< 0.01
TG, mmol/L	1.13 (0.85–1.61)	1.21 (0.84–1.85)	1.28 (0.95–1.82)	1.45 (1.06–2.09)	< 0.01
LDL-C, mmol/L	1.46 (1.19–1.64)	1.45 (1.17–1.63)	2.58 (2.16–3.19)	2.72 (2.25–3.22)	< 0.01
HDL-C, mmol/L	1.15 (0.99–1.42)	1.17 (0.98–1.45)	1.30 (1.11–1.55)	1.23 (1.05–1.46)	< 0.01
SBP, mmHg	139.23 ± 19.66	140.26 ± 19.98	141.49 ± 20.37	144.21 ± 20.63	< 0.01
FBG, mmol/L	5.79 (5.12–6.77)	5.69 (5.04–6.64)	5.70 (5.11–6.96)	5.90 (5.20–7.40)	< 0.01
hs-CRP, mg/L	0.73 (0.44–1.30)	3.80 (2.60–5.42)	0.80 (0.45–1.21)	3.70 (2.60–5.10)	< 0.01
High school education or above, n (%)	109 (17.8)	39 (13.2)	267 (16.4)	172 (17.7)	0.28
Alcohol drinking, n (%)	100 (16.3)	51 (17.3)	312 (19.2)	171 (17.6)	0.41
Smoking, n (%)	116 (18.9)	62 (21.0)	314 (19.3)	203 (20.9)	0.67
Salt intake > 10 g/day, n (%)	33 (5.4)	11 (3.7)	94 (5.8)	60 (6.2)	0.45
Physical activity, n (%)	370 (60.4)	177 (60.0)	1090 (67.0)	616 (63.3)	< 0.01
Risk score, %	28 (19–40)	32 (23–45)	31 (22–45)	37 (28–52)	< 0.01
Hypertension, n (%)	530 (86.5)	266 (90.2)	1443 (88.6)	893 (91.8)	< 0.01
Diabetes mellitus, n (%)	153 (25.0)	70 (23.7)	467 (28.7)	319 (32.8)	< 0.01
Antihypertensive medication use, n (%)	445 (72.6)	227 (76.9)	1209 (74.3)	750 (77.1)	0.16
Antidiabetic medication use, n (%)	66 (10.8)	29 (9.8)	199 (12.2)	129(13.3)	0.29
Antiplatelet medication use, n (%)	254 (41.4)	126 (42.7)	580 (35.6)	343 (35.3)	< 0.01
Properties of statins					0.03
Lipophilic	367(59.9)	181 (61.4)	1058 (65.0)	644 (66.2)	
Hydrophilic	35 (5.7)	9(3.1)	90 (5.5)	42 (4.3)	
Both of all	211 (34.4)	105(35.6)	480 (29.5)	287 (29.5)	
MPR ≥ 80%	100 (16.3)	40 (13.6)	338 (20.8)	198 (20.3)	< 0.01

Abbreviations: BMI, body mass index; DBP, diastolic blood pressure; FBG, fasting blood glucose; HDL-C, high-density lipoprotein cholesterol; hs-CRP, high-sensitivity C-reactive protein; LDL-C, low-density lipoprotein cholesterol; MPR, medication possession ratio; RCIR, residual cholesterol and inflammatory risk; RCR, residual cholesterol risk; RIR, residual inflammatory risk; SBP, systolic blood pressure; TC, total cholesterol; TG, triglycerides.

**Table 2:** Incidence Density and Hazard Ratio (95% Confidence Interval) of All-cause Mortality in Each Group

	No residual risk	RIR	RCR	RCIR	
Case/Participants, n/n	46/613	36/295	166/1628	129/973	
Incidence (/1,000 person years)	12.62 (9.46, 16.85)	20.56 (14.84, 28.51)	18.13 (15.57, 21.11)	23.12 (19.46, 27.48)	
Model 1	1.00	1.65 (1.07, 2.55)	1.48 (1.07, 2.05)	1.85 (1.32, 2.59)	
Model 2	1.00	1.64 (1.06, 2.54)	1.38 (0.99, 1.92)	1.75 (1.24, 2.45)	
Model 3	1.00	1.66 (1.07, 2.57)	1.36 (0.98, 1.89)	1.74 (1.24, 2.45)	
Model 4	1.00	1.65 (1.07, 2.56)	1.33 (0.96, 1.85)	1.72 (1.22, 2.42)	

Model 1: adjusted for age, sex; Model 2: on the basis of model 1, adjusted for educational background, physical activity, smoking status, drinking status, salt intake, BMI, hypertension, diabetes mellitus, history of CVD, properties of statins; Model 3: on the basis of model 2, adjusted for antihypertensive medication use, antidiabetic medication use, antiplatelet medication use; Model 4: on the basis of model 3, adjusted for TG, HDL-C.

Abbreviations: RCIR, residual cholesterol and inflammatory risk; RCR, residual cholesterol risk; RIR, residual inflammatory risk.

### Association between residual risk and all-cause mortality

During a median follow-up of 6.10 (2.96, 8.47) years, 377 deaths occurred. The number of deaths and incidence density in each group are shown in Table 2. We conducted a log-rank test with p < 0.01 for the four groups as a whole (Additional file 1: Figure S2). The association between residual risk and all-cause mortality remained after adjustment for all covariates in all study participants. Compared with no residual risk, the HR (95% CI) of all-cause mortality for RIR, RCR, and RCIR were 1.65 (1.05, 2.56), 1.33 (0.96, 1.85), and 1.72 (1.22, 2.42), respectively, as shown in Table 2. The results were consistent with the main results after excluding 50 participants who died or were followed for less than 1 year. The results were stable when the critical values of hs-CRP and LDL-C were changed to 3 mg/L and 2.6 mmol/L, respectively, and when the critical values of hs-CRP and LDL-C were changed simultaneously (Additional file 2: Table S1).

**Fig. 1:**
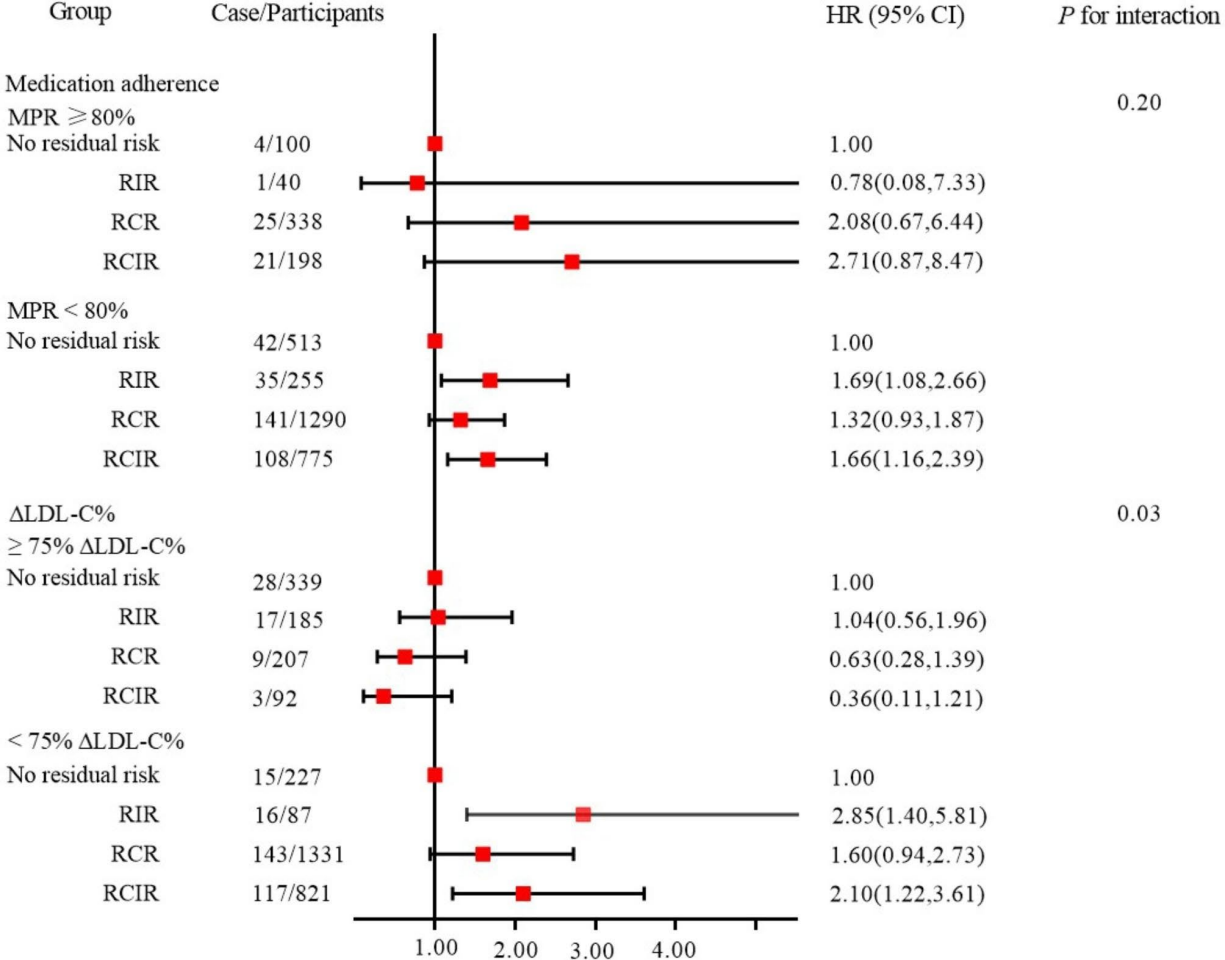
Hazard ratio and 95% confidence interval for participants with different degrees of medication adherence and reductions in low-density lipoprotein cholesterol Abbreviations: MPR, medication possession ratio; RCIR, residual cholesterol and inflammatory risk; RCR, residual cholesterol risk; RIR, residual inflammatory risk. ∆LDL-C% was defined as the ratio of the difference between the most recent LDL-C measurement before baseline and the baseline LDL-C to the most recent LDL-C measurement before baseline.

The HR and 95% CI of all-cause mortality with RCIR and RIR were 1.69 (1.09, 2.66) and 1.66 (1.16, 2.39), respectively, in the non-adherence group. No significant difference was found for the other three groups within the group with good adherence. For the remaining 3289 patients in the stratified analysis of ∆LDL-C% (177 did not undergo the most recent physical examination before receiving medication, and 43 were missing LDL-C data), the results showed that the HR and 95% CI of all-cause mortality with RIR and RCIR in the group with < 75% ∆LDL-C% were 2.85 (1.40, 5.81) and 2.10 (1.22, 3.61), respectively. For the other three groups, ≥ 75% ∆LDL-C% showed no significant difference (Fig. 1; Additional file 2: Table S2).

**Fig. 2:**
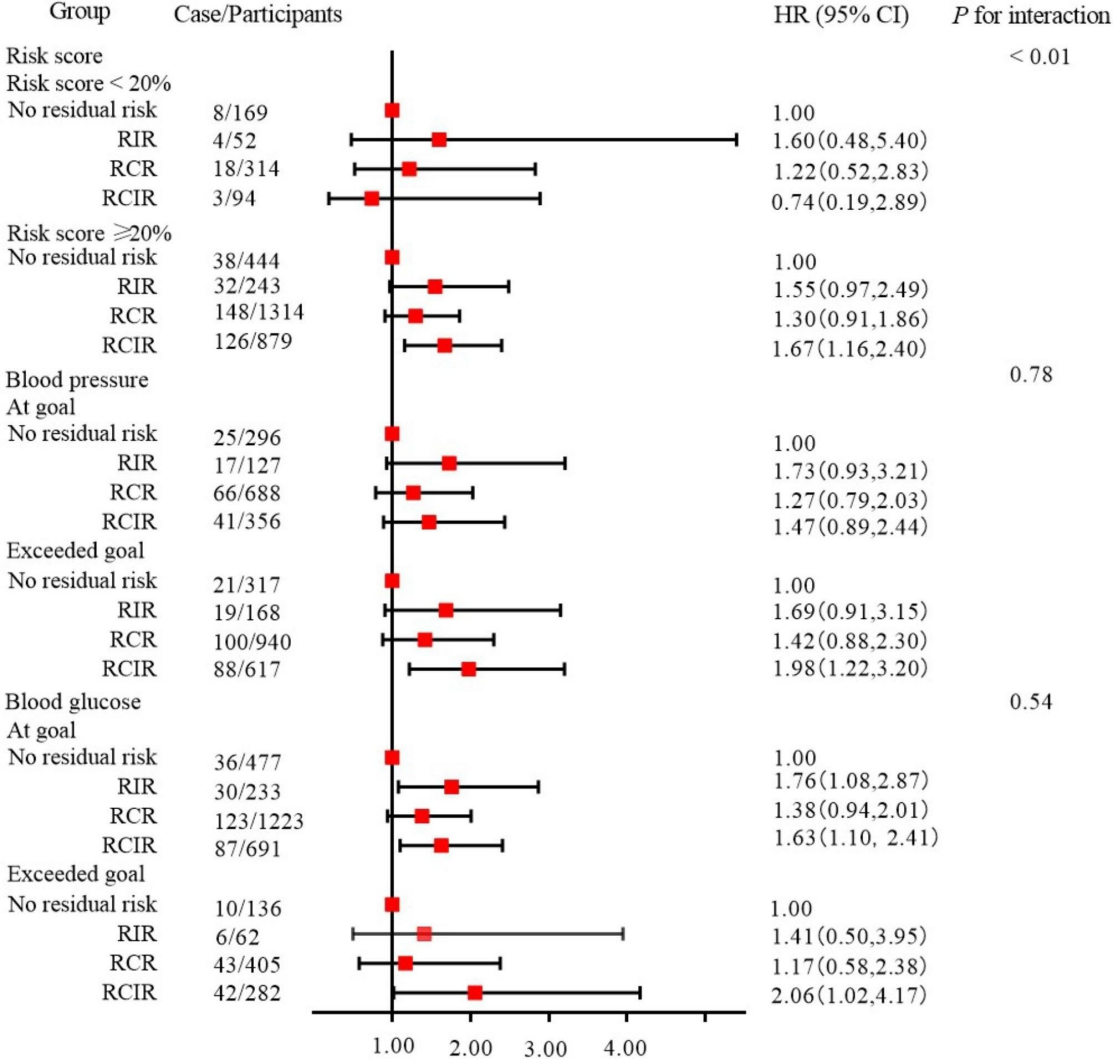
Hazard ratio and 95% confidence interval for participants with different risk scores Abbreviations: RCIR, residual cholesterol and inflammatory risk; RCR, residual cholesterol risk; RIR, residual inflammatory risk. Blood pressure: At goal: SBP < 140 mmHg and DBP < 90 mmHg; Exceeded goal: SBP ≥ 140 mmHg or DBP ≥ 90 mmHg. Blood glucose: At goal: FBG < 7.0 mmol/L; Exceeded goal: FBG ≥ 7.0 mmol/L.

When stratified by SMART 2 risk score, only the RCIR with a SMART 2 risk score ≥ 20% had a significantly increased risk of all-cause mortality (HR 1.67; 95% CI 1.16, 2.40). There was no significant increase in the risk of all-cause mortality compared with no residual risk in the remaining three groups among those with a SMART 2 risk score < 20%. When stratified according to whether participants in normal blood pressure, the risk of all-cause mortality was significantly increased among those with uncontrolled blood pressure (HR 1.98; 95% CI 1.22, 3.20). There was no significant increase in the risk of all-cause mortality compared with no residual risk in the remaining three groups that had reached the blood pressure targets. Stratified analysis was conducted according to whether participants in normal blood glucose, RCIR (HR 2.06; 95% CI 1.02, 4.17) was associated with a higher risk of all-cause mortality among those with uncontrolled blood glucose. Among those with controlled blood glucose, the RIR (HR 1.76; 95% CI 1.08, 2.87) and RCIR (HR 1.63; 95% CI 1.10, 2.41) showed a higher risk of all-cause mortality (Fig. 2; Additional file 2: Table S3).

The results of interaction between LDL-C and hs-CRP showed that multiplicative interaction is not significant (HR 0.98; 95%CI 0.94, 1.03). All the indexes of additive interaction were not statistically significant (RERI − 0.26, 95%CI -1.00, 0.49; AP -0.15, 95%CI -0.54, 0.25; SI 0.74, 95%CI 0.35, 1.56).

## Discussion

We found that in our real-world study population, patients with CVD and simultaneous risks of residual cholesterol and residual inflammation had an increased risk of mortality. The increased risk was dependent on adherence to statins, reduction in LDL-C, SMART 2 risk score, and control of blood pressure and blood glucose. Our results verified the consistency between real-world and RCT results.

We found that the risk of all-cause mortality in the RCIR was increased by 72% compared with the group that had no residual risk during a follow-up of 6.10 years. Even after adjusting critical values of LDL-C (from 1.8 mmol/L to 2.6 mmol/L) and hs-CRP (from 2 mg/L to 3 mg/L), the risk of all-cause mortality in the RCIR still increased by more than 75%. The increase in relative risk was greater than those of cholesterol and inflammation alone, suggesting that residual cholesterol and residual inflammatory risk may have a combined effect. Multiple RCTs have found a 37–390% increased risk of MACE in people with residual risk of cholesterol and inflammation compared with those who have no residual risk [9, 22, 23]. However, the longest follow-up period in the aforementioned studies was no more than 5 years. Only one real-world study found that with LDL-C ≥ 1.42 mmol/L in patients with ischemic stroke, hs-CRP ≥ 2 mg/L was associated with a higher risk of stroke recurrence [24]. However, that study only collected data for stroke recurrence within 1 year after follow-up. Ours is the first real-world study to explore the association between the dual risks of cholesterol and inflammation and all-cause mortality.

In this study, we also found a positive association between the dual risk of cholesterol and inflammation and all-cause mortality, which showed a dependence on statin adherence and LDL-C reduction. The risk of all-cause mortality was 66% higher in the RCIR than in the group with no residual risk for participants with moderate or low adherence, and no increase was observed in participants with good adherence. Consistent with our results, among patients with previous atherosclerotic cardiovascular disease (ASCVD), those who had the lowest compliance with statins (MPR < 50%) had a 1.30-times higher risk of mortality than those with good compliance (70%<MPR < 89%) [19]. Compared with the group with no residual risk, we observed a 110% increase in the risk of all-cause mortality in the RCIR with a low reduction in LDL-C. In contrast, no increase in the risk of all-cause mortality was observed in the group with a higher LDL-C reduction. A meta-analysis showed that with baseline LDL-C > 2.6 mmol/L, the risk of all-cause mortality gradually decreased with an increased reduction in LDL-C [25]. A recent study also found that patients with myocardial infarction who had a significant decrease in LDL-C in the early stage had a 29% lower risk of all-cause mortality [26]. Given the poor adherence of Asian populations to long-term statin use [27], only one in five statin users in our study had good compliance. Therefore, it is necessary for clinicians and health systems to eliminate the residual risk of cholesterol and inflammation in patients with CVD by closely following statin adherence, testing LDL-C levels at specific intervals, and regularly discussing ways to improve statin adherence, including adherence to medication and a healthy lifestyle.

We found that controlling risk factors eliminated the dual risk of cholesterol and inflammation. Among people with a SMART 2 risk score ≥ 20%, the risk of all-cause mortality was 1.67-times higher in participants with RCIR than in those with no residual risk. Among those whose blood pressure was not controlled, participants with RCIR had 1.98 times the risk of all-cause mortality compared with participants who had no residual risk. However, no association between residual risk and mortality was found in people with a SMART 2 risk score < 20% and those with compliant blood pressure. An RCT found that the cumulative incidence of MACE in high-risk patients with CVD (acute coronary syndrome, stroke, peripheral arterial disease, or type 2 diabetes with coronary artery disease in the past 30 to 365 days) treated with statins increased with increasing lipid levels when hs-CRP was ≥ 2 mg/L. However, this association was not found in patients with hs-CRP < 2 mg/L [28]. Additionally, in participants with uncontrolled blood glucose, we observed that the risk of all-cause mortality was 2.06-times higher in participants with RCIR than in those with no residual risk. The mortality risk was 1.63 times higher in the normal blood glucose participants with RCIR than in those with no residual risk, which was lower than that with RCIR when blood glucose was not up to standard. Therefore, in addition to actively controlling levels of cholesterol and inflammation, patients with CVD should also address a variety of risk factors, including blood pressure, blood glucose, and risk factors involved in the SMART 2 risk score.

LDL-C and hs-CRP play vital roles in the recurrence and development of ASCVD, and ASCVD and its complications are leading causes of mortality. High levels of LDL-C can accumulate in the intima of the artery, carry residual cholesterol, and can then be oxidized [29]. Oxidized LDL-C (Ox-LDL-C) can stimulate endothelial cells and macrophages, cause endothelial dysfunction, lead to atherosclerosis, and increase the mortality risk [30]. Ox-LDL-C can also increase the expression of hs-CRP [31], which can activate the complement system to form atherosclerotic plaque [32, 33]. The higher the hs-CRP level, the more unstable the plaque, which can easily cause ASCVD. Additionally, many studies have demonstrated an association between hs-CRP and a range of diseases, such as hypertension, coronary artery disease, stroke, and cancer, which significantly contribute to death [34–37]. Additionally, hs-CRP can increase LDL-C transport between endothelial cells and increase the expression of Ox-LDL-C, thereby forming a positive feedback loop between LDL-C and hs-CRP, which jointly increase the risk of mortality [38].

Among real-world studies on the dual risk of cholesterol and inflammation, our study had the longest follow-up time, and the data used were from the Kailuan Study. Drug prescription information was collected from the chronic disease clinic of the hospital, and death events were determined using the medical information system and social security system, which are reliable data. Statin costs for patients with CVD are reimbursed at 80% through employee health insurance, so statin adherence is less affected by the ability to pay. Additionally, our study is based on real-world data, and the study participants were broadly representative, for patients with CVD with a number of coexisting comorbidities and drug therapies. However, this study has the following limitations. First, this was a single-center study with 86.78% men, so generalization of the results may be limited. Second, we did not collect statin dosage information; further exploration of the association between residual risk and mortality at different doses is needed. Third, data from a single measurement of LDL-C and hs-CRP do not reflect average levels during long-term follow-up. Fourth, there is a lack of information on the causes of mortality and failure to distinguish between CVD causes and non-CVD causes.

## Conclusion

The dual residual risk of cholesterol and inflammation significantly increased the risk of all-cause mortality in patients with CVD who were treated with statins. This increased risk was dependent on statin adherence, LDL-C reduction, SMART 2 risk score, and control of blood pressure and blood glucose. Therefore, in addition to receiving long-term and effective combined lipid-lowering and anti-inflammatory therapy, it is recommended that patients with CVD should improve statin compliance and control multiple risk factors to reduce the risk of mortality.

## Electronic supplementary material

Below is the link to the electronic supplementary material.

Additional file 1: figure S1
 Flowchart of the study. Figure S2. Log-rank test of all groups.

Additional file 2: table S1
 Sensitivity Analysis. Table S1. Hazard Ratio and 95% Confidence Interval for Participants with Different Degrees of Medication Adherence and Reductions in Low-density Lipoprotein Cholesterol. Table S3. Hazard Ratio and 95% Confidence Interval for Participants with Different Risk Scores. Table S4. Interaction effect of LDL-C and hs-CRP on the risk of all-cause mortality.

## Data Availability

The datasets used and analyzed during the current study are available from the corresponding author on reasonable request.

## Abbreviations

ASCVD: atherosclerotic cardiovascular disease
ICD: International Classification of Diseases
MACE: major adverse cardiovascular events
PH: proportional hazards
RCIR: residual cholesterol and inflammatory risk
RCR: residual cholesterol risk
RIR: residual inflammatory risk
