# Supplementary material for: Effect of dual residual risk of cholesterol and inflammation on all-cause mortality in patients with cardiovascular disease
Source: Cardiovasc Diabetol. 2023 Apr 24;22:96. doi: 10.1186/s12933-023-01826-3 (PMC10127069; doi:10.1186/s12933-023-01826-3)
Supplement: Supplementary file 1 — Additional file 1: figure S1 Flowchart of the study. Figure S2. Log-rank test of all groups. [file 12933_2023_1826_MOESM1_ESM.docx]

**Supplemental materials**

Effect of dual residual risk of cholesterol and inflammation on all-cause mortality in patients with cardiovascular disease

Ling Yang, PhD et al.

Figure S1. Flowchart of the study

Figure S2. Log-rank test of all groups


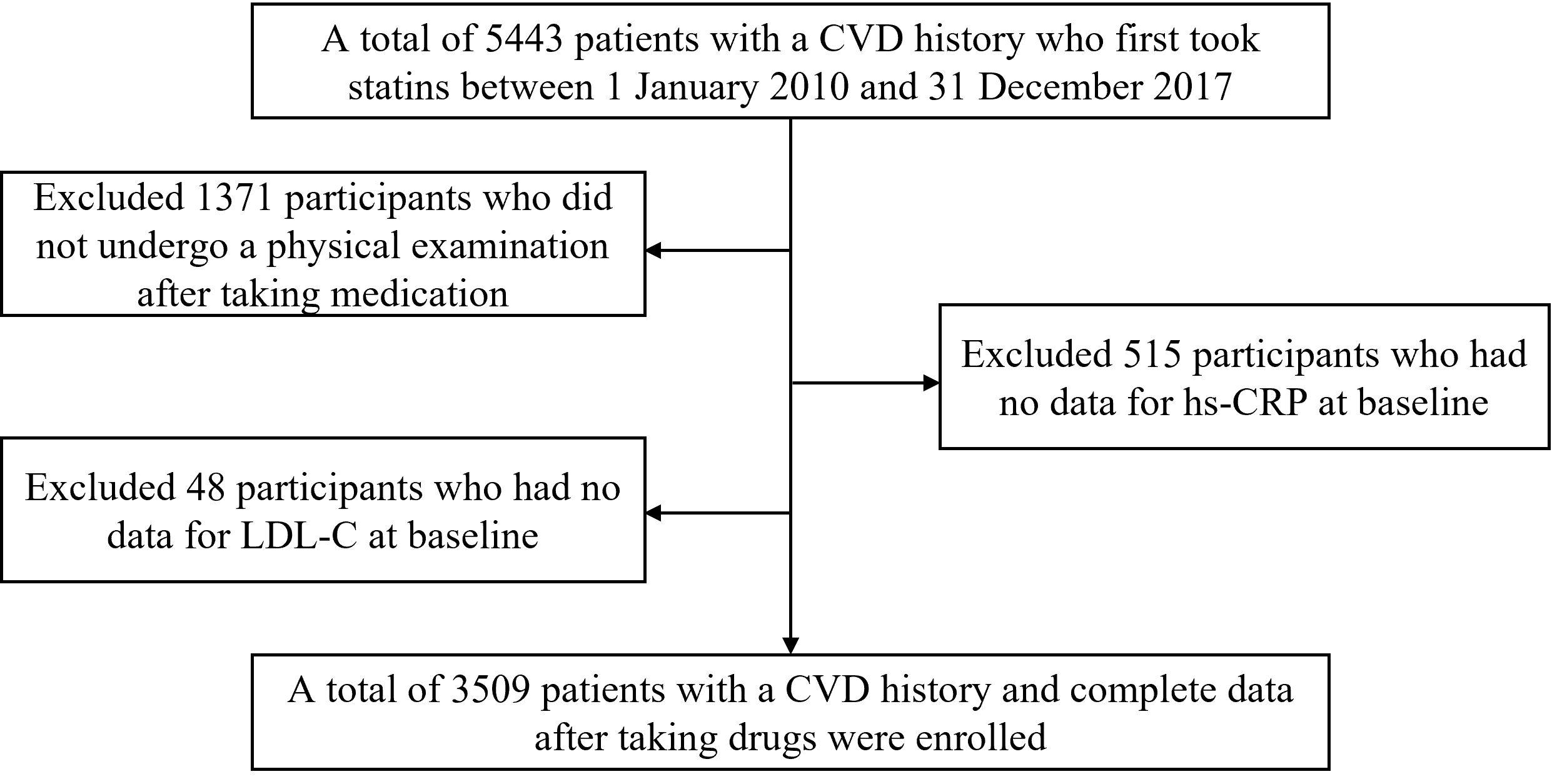


Figure S1. Flowchart of the study


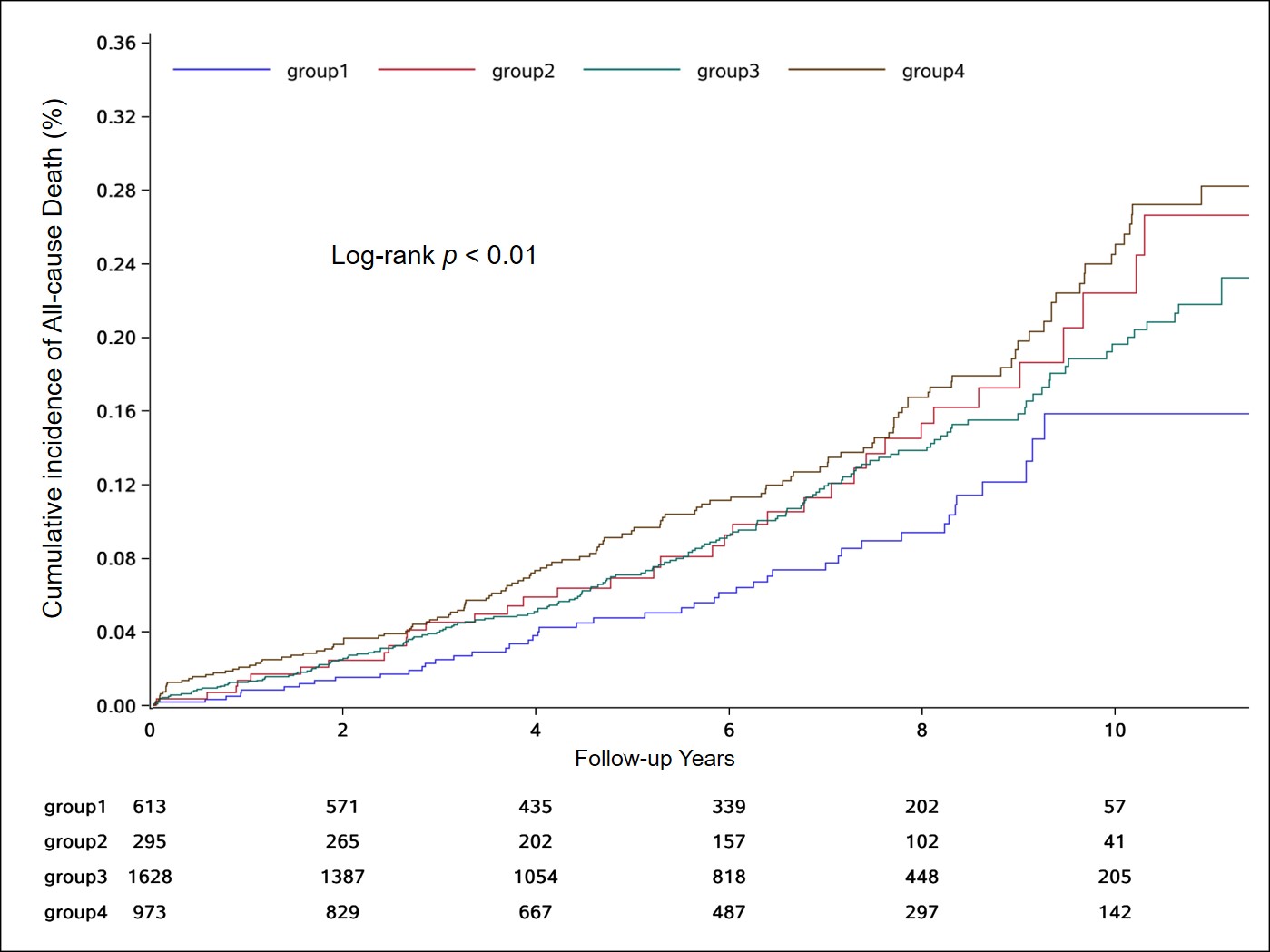


Figure S2. Log-rank test of all group
