# Supplementary material for: Effect of dual residual risk of cholesterol and inflammation on all-cause mortality in patients with cardiovascular disease
Source: Cardiovasc Diabetol. 2023 Apr 24;22:96. doi: 10.1186/s12933-023-01826-3 (PMC10127069; doi:10.1186/s12933-023-01826-3)
Supplement: Supplementary file 2 — Additional file 2: table S1 Sensitivity Analysis. Table S1. Hazard Ratio and 95% Confidence Interval for Participants with Different Degrees of Medication Adherence and Reductions in Low-density Lipoprotein Cholesterol. Table S3. Hazard Ratio and 95% Confidence Interval for Participants with Different Risk Scores. Table S4. Interaction effect of LDL-C and hs-CRP on the risk of all-cause mortality. [file 12933_2023_1826_MOESM2_ESM.docx]

**Supplemental materials**

Effect of dual residual risk of cholesterol and inflammation on all-cause mortality in patients with cardiovascular disease

Ling Yang, et al.

Table S1. Sensitivity Analysis

Table S2. Hazard Ratio and 95% Confidence Interval for Participants with Different Degrees of Medication Adherence and Reductions in Low-density Lipoprotein Cholesterol

Table S3. Hazard Ratio and 95% Confidence Interval for Participants with Different Risk Scores

Table S4. Interaction effect of LDL-C and hs-CRP on the risk of all-cause mortality

| Table S1. Sensitivity Analysis | | | | |
| --- | --- | --- | --- | --- |
|  | No residual risk | RIR | RCR | RCIR |
| Sensitivity 1 |  |  |  |  |
| Case/Participants, n/n | 41/607 | 32/291 | 146/1608 | 109/953 |
| Incidence (/1,000 person years) | 11.26 (8.29, 15.30) | 18.31 (12.95, 25.89) | 15.96 (13.57, 18.77) | 19.56 (16.21, 23.60) |
| Model 4 | 1.00 | 1.64 (1.03, 2.61) | 1.32 (0.93, 1.87) | 1.63 (1.13, 2.35) |
| Sensitivity 2 |  |  |  |  |
| Case/Participants, n/n | 53/708 | 29/200 | 209/1961 | 86/640 |
| Incidence (/1,000 person years) | 12.52 (9.56, 16.38) | 25.00 (17.37, 35.98) | 18.75 (16.37, 21.47) | 23.95 (19.39, 29.59) |
| Model 4 | 1.00 | 2.09 (1.33, 3.30) | 1.42 (1.04, 1.93) | 1.75 (1.24, 2.47) |
| Sensitivity 3 |  |  |  |  |
| Case/Participants, n/n | 117/1440 | 77/722 | 95/801 | 88/546 |
| Incidence (/1,000 person years) | 14.26 (11.89, 17.09) | 18.41 (14.73, 23.02) | 20.67 (16.91, 25.28) | 27.97 (22.69, 34.45) |
| Model 4 | 1.00 | 1.33 (0.99, 1.77) | 1.36 (1.03, 1.78) | 1.84 (1.39, 2.43) |
| Sensitivity 4 |  |  |  |  |
| Case/Participants, n/n | 46/1677 | 36/485 | 166/992 | 129/355 |
| Incidence (/1,000 person years) | 12.62 (9.46, 16.85) | 20.57 (15.57, 21.11) | 18.13 (15.57, 21.11) | 23.12 (19.46, 27.48) |
| Model 4 | 1.00 | 1.50 (1.10, 2.04) | 1.46 (1.14, 1.87) | 1.84 (1.34, 2.53) |
| Sensitivity 1: excluding 50 participants who died within one year or follow-up time less than one year, the remaining 3459 participants on the basis of Model 4; Sensitivity 2: Change hs-CRP to 3 mg/L with a cut-off of 2 mg/L on the basis of Model 4. Sensitivity 3: Change LDL-C to 2.6 mmol/L with a cut-off of 1.8 mmol/L on the basis of Model 4. Sensitivity 4: Change LDL-C to 2.6 mmol/L with a cut-off of 1.8 mmol/L and Change hs-CRP to 3 mg/L with a cut-off of 2 mg/L on the basis of Model 4.  Abbreviations: RCIR, residual cholesterol and inflammatory risk; RCR, residual cholesterol risk; RIR, residual inflammatory risk. | | | | |

| Table S2. Hazard Ratio and 95% Confidence Interval for Participants with Different Degrees of Medication Adherence and Reductions in Low-density Lipoprotein Cholesterol | | | | | |  |
| --- | --- | --- | --- | --- | --- | --- |
|  | No residual risk | RIR | RCR | RCIR | *P* for interaction | |
| Medication adherence |  |  |  |  | 0.20 | |
| MPR ≥ 80% |  |  |  |  |  | |
| Case/Participants, n/n | 4/100 | 1/40 | 25/338 | 21/198 |  | |
| Incidence (/1,000 person years) | 10.18 (3.82, 27.12) | 6.03 (0.85, 42.80) | 18.60 (12.57, 27.53) | 24.98 (16.29, 38.32) |  | |
| HR (95% CI)* | 1.00 | 0.78 (0.08, 7.33) | 2.08 (0.67, 6.44) | 2.71 (0.87, 8.47) |  | |
| MPR < 80% |  |  |  |  |  | |
| Case/Participants, n/n | 42/513 | 35/255 | 141/1290 | 108/775 |  | |
| Incidence (/1,000 person years) | 12.92 (9.55, 17.48) | 22.09 (15.86, 30.77) | 18.05 (15.30, 21.28) | 22.79 (18.88, 27.52) |  | |
| HR (95% CI)* | 1.00 | 1.69 (1.08, 2.66) | 1.32 (0.93, 1.87) | 1.66 (1.16, 2.39) |  | |
| ∆LDL-C% |  |  |  |  | 0.03 | |
| ≥ 75% ∆LDL-C% |  |  |  |  |  | |
| Case/Participants, n/n | 28/339 | 17/185 | 9/207 | 3/92 |  | |
| Incidence (/1,000 person years) | 13.81 (9.53, 20.00) | 15.32 (9.52, 24.64) | 9.53 (4.96, 18.31) | 5.98 (1.93, 18.54) |  | |
| HR (95% CI) | 1.00 | 1.04 (0.56, 1.96) | 0.63 (0.28, 1.39) | 0.36 (0.11, 1.21) |  | |
| < 75% ∆LDL-C% |  |  |  |  |  | |
| Case/Participants, n/n | 15/227 | 16/87 | 143/1331 | 117/821 |  | |
| Incidence (/1,000 person years) | 11.36 (6.85, 18.84) | 31.21 (19.12, 50.94) | 18.66 (15.84, 21.98) | 24.77 (20.67, 29.69) |  | |
| HR (95% CI) | 1.00 | 2.85 (1.40, 5.81) | 1.60 (0.94, 2.73) | 2.10 (1.22, 3.61) |  | |
| Adjusted on the basis of model 4 + Interval time between two LDL-C measurements  Abbreviations: MPR, medication possession ratio; RCIR, residual cholesterol and inflammatory risk; RCR, residual cholesterol risk; RIR, residual inflammatory risk. ∆LDL-C% was defined as the ratio of the difference between the most recent LDL-C measurement before baseline and the baseline LDL-C to the most recent LDL-C measurement before baseline. | | | | | |  |

| Table S3. Hazard Ratio and 95% Confidence Interval for Participants with Different Risk Scores | | | | | |
| --- | --- | --- | --- | --- | --- |
|  | No residual risk | RIR | RCR | RCIR | *P* for interaction |
| Risk score |  |  |  |  | < 0.01 |
| Risk score < 20% |  |  |  |  |  |
| Case/Participants, n/n | 8/169 | 4/52 | 18/314 | 3/94 |  |
| Incidence (/1,000 person years) | 7.17 (3.59, 14.34) | 9.97 (3.74, 26.56) | 9.09 (5.72, 14.42) | 4.32 (1.39, 13.40) |  |
| HR (95% CI)^#^ | 1.00 | 1.60 (0.48, 5.40) | 1.22 (0.52, 2.83) | 0.74 (0.19, 2.89) |  |
| Risk score ≥ 20% |  |  |  |  |  |
| Case/Participants, n/n | 38/444 | 32/243 | 148/1314 | 126/879 |  |
| Incidence (/1,000 person years) | 15.03 (10.93, 20.65) | 23.72 (16.77, 33.54) | 20.62 (17.55, 24.23) | 25.79 (21.66, 30.72) |  |
| HR (95% CI)^#^ | 1.00 | 1.55 (0.97, 2.49) | 1.30 (0.91, 1.86) | 1.67 (1.16, 2.40) |  |
| Blood pressure |  |  |  |  | 0.78 |
| At goal |  |  |  |  |  |
| Case/Participants, n/n | 25/296 | 17/127 | 66/688 | 41/356 |  |
| Incidence (/1,000 person years) | 13.63 (9.21, 20.18) | 22.37 (13.91, 35.99) | 17.72 (13.92, 22.56) | 20.28 (14.93, 27.55) |  |
| HR (95% CI)* | 1.00 | 1.73 (0.93, 3.21) | 1.27 (0.79, 2.03) | 1.47 (0.89, 2.44) |  |
| Exceeded goal |  |  |  |  |  |
| Case/Participants, n/n | 21/317 | 19/168 | 100/940 | 88/617 |  |
| Incidence (/1,000 person years) | 11.60 (7.56, 17.79) | 19.18 (12.24, 30.08) | 18.43 (15.13, 22.39) | 24.74 (15.13, 22.39) |  |
| HR (95% CI)* | 1.00 | 1.69 (0.91, 3.15) | 1.42 (0.88, 2.30) | 1.98 (1.22, 3.20) |  |
| Blood glucose |  |  |  |  | 0.54 |
| At goal |  |  |  |  |  |
| Case/Participants, n/n | 36/477 | 30/233 | 123/1223 | 87/691 |  |
| Incidence (/1,000 person years) | 12.42 (8.96, 17.21) | 21.36 (14.94, 30.56) | 17.59 (14.74, 20.98) | 20.94 (16.97, 25.84) |  |
| HR (95% CI)* | 1.00 | 1.76 (1.08, 2.87) | 1.38 (0.94, 2.01) | 1.63 (1.10, 2.41) |  |
| Exceeded goal |  |  |  |  |  |
| Case/Participants, n/n | 10/136 | 6/62 | 43/405 | 42/282 |  |
| Incidence (/1,000 person years) | 13.43 (7.23, 24.97) | 17.34 (7.79, 38.58) | 19.88 (14.74, 26.51) | 29.47 (21.78, 39.58) |  |
| HR (95% CI)* | 1.00 | 1.41 (0.50, 3.95) | 1.17 (0.58, 2.38) | 2.06 (1.02, 4.17) |  |
| #: Using the SMART 2 risk score to calculate the risk score, and adjusted for educational background , physical activity, drinking status, salt intake, BMI, antihypertensive medication use, TG  *: Adjusted on the basis of model 4.  Abbreviations: RCIR, residual cholesterol and inflammatory risk; RCR, residual cholesterol risk; RIR, residual inflammatory risk.  Blood pressure: At goal: SBP < 140 mmHg and DBP < 90 mmHg; Exceeded goal: SBP ≥ 140 mmHg or DBP ≥ 90 mmHg.  Blood glucose: At goal: FBG < 7.0 mmol/L; Exceeded goal: FBG ≥ 7.0 mmol/L. | | | | | |

| Table S4. Interaction effect of LDL-C and hs-CRP on the risk of all-cause mortality | | | | |
| --- | --- | --- | --- | --- |
|  | Additive interaction (95% CI) | | | Multiplicative interaction (95% CI) |
|  | RERI | AP | SI |  |
| Model 1 | -0.21 (-0.94, 0.52) | -0.12 (-0.50, 0.26) | 0.79 (0.39, 1.31) | 0.98 (0.94, 1.02) |
| Model 2 | -0.28 (-1.04, 0.49) | -0.15 (-0.54, 0.25) | 0.76 (0.39, 1.47) | 0.98 (0.94, 1.02) |
| Model 3 | -0.26 (-1.01, 0.49) | -0.15 (-0.55, 0.25) | 0.74 (0.36, 1.53) | 0.98 (0.94, 1.03) |
| Model 4 | -0.26 (-1.00, 0.49) | -0.15 (-0.54, 0.25) | 0.74 (0.35, 1.56) | 0.98 (0.94, 1.03) |
| Model 1：adjusted for age, sex; Model 2：on the basis of model 1, adjusted for educational background, physical activity, smoking status, drinking status, salt intake, BMI, hypertension, diabetes mellitus, history of CVD, properties of statins; Model 3：on the basis of model 2, adjusted for antihypertensive medication use, antidiabetic medication use, antiplatelet medication use; Model 4：on the basis of model 3, adjusted for TG, HDL-C.  RERI= HR _RCIR_ - HR _RCR_ - HR _RIR_ +1;  AP= (HR _RCIR_ - HR _RCR_ - HR _RIR_ +1) / HR _RCIR_;  SI= (HR _RCIR_ -1) / (HR _RCR_ + HR _RIR_ -2)  Abbreviations: RERI, relative excess risk due to interaction; AP, attributable proportion due to interaction; SI, synergy index. | | | | |
